# PBRM1 loss is associated with increased sensitivity to MCL1 and CDK9 inhibition in clear cell renal cancer

**DOI:** 10.3389/fonc.2024.1343004

**Published:** 2024-02-02

**Authors:** Norman Fultang, Ashley M. Schwab, Sophia McAneny-Droz, Alexander Grego, Stephanie Rodgers, Brian Vidal Torres, Diane Heiser, Peggy Scherle, Neha Bhagwat

**Affiliations:** Prelude Therapeutics Inc, Wilmington, DE, United States

**Keywords:** MCL1, PBRM1, renal cancer, PRT1419, CDK9

## Abstract

MCL1 is a member of the BCL2 family of apoptosis regulators, which play a critical role in promoting cancer survival and drug resistance. We previously described PRT1419, a potent, MCL1 inhibitor with anti-tumor efficacy in various solid and hematologic malignancies. To identify novel biomarkers that predict sensitivity to MCL1 inhibition, we conducted a gene essentiality analysis using gene dependency data generated from CRISPR/Cas9 cell viability screens. We observed that clear cell renal cancer (ccRCC) cell lines with damaging *PBRM1* mutations displayed a strong dependency on MCL1. PBRM1 (BAF180), is a chromatin-targeting subunit of mammalian pBAF complexes. PBRM1 is frequently altered in various cancers particularly ccRCC with ~40% of tumors harboring damaging PBRM1 alterations. We observed potent inhibition of tumor growth and induction of apoptosis by PRT1419 in various preclinical models of PBRM1-mutant ccRCC but not PBRM1-WT. Depletion of PBRM1 in PBRM1-WT ccRCC cell lines induced sensitivity to PRT1419. Mechanistically, PBRM1 depletion coincided with increased expression of pro-apoptotic factors, priming cells for caspase-mediated apoptosis following MCL1 inhibition. Increased MCL1 activity has been described as a resistance mechanism to Sunitinib and Everolimus, two approved agents for ccRCC. PRT1419 synergized with both agents to potently inhibit tumor growth in PBRM1-loss ccRCC. PRT2527, a potent CDK9 inhibitor which depletes MCL1, was similarly efficacious in monotherapy and in combination with Sunitinib in PBRM1-loss cells. Taken together, these findings suggest PBRM1 loss is associated with MCL1i sensitivity in ccRCC and provide rationale for the evaluation of PRT1419 and PRT2527 for the treatment for PBRM1-deficient ccRCC.

## 1 Introduction

Induced myeloid leukemia cell differentiation protein (MCL1) is a crucial member of the B-cell lymphoma-2 (BCL2) family of apoptosis regulators, playing a significant role in maintaining cellular homeostasis and promoting cancer cell survival (1, 2). Aberrant expression of MCL1 has been linked to poor prognosis and resistance to chemotherapeutic and targeted agents in various cancers (3–6). In these malignancies, pharmacological inhibition of MCL1 has demonstrated potential to inhibit tumor growth and restore sensitivity to standard of care agents (S.o.C) (7–9). PRT1419 is a potent and selective MCL1 inhibitor with anti-tumor efficacy in diverse preclinical models of solid and hematologic malignancies, which is currently being evaluated in a Phase 1 clinical trial (NCT05107856).

To improve clinical outcomes, there remains a pressing need to identify genomic biomarkers that can predict sensitivity to MCL1 inhibition to aid patient selection. To this end, we conducted a gene essentiality analysis using publicly available human cancer cell line gene dependency data from genome wide CRISPR/Cas9 cell viability screens (10, 11). Our analysis revealed that clear cell Renal Cell Carcinoma (ccRCC) cell lines with deleterious alterations in the *PBRM1* (Polybromo 1) gene displayed a strong dependency on MCL1. PBRM1, also known as BAF180, is a crucial chromatin-targeting subunit of mammalian pBAF (SWI/SNF-B) complexes (12, 13). Frequent alterations in PBRM1 have been observed across various human cancers, but its alteration rate is particularly high in ccRCC, with approximately 40% of tumors harboring damaging PBRM1 alterations (12).

ccRCC is a histological subtype of renal cancer (RC) representing ~70-75% of all new RC diagnoses (14, 15). Most newly diagnosed ccRCC cases are localized and are primarily treated with surgical resection or nephrectomy (16). While these options are curative in most cases, approximately 30% of all patients develop recurrent disease within 5 years (16). In patients with recurrent or metastatic disease, options are limited, and overall survival rates are poor (17). For patients with metastatic ccRCC, approved therapies include cytokine therapy, tyrosine kinase inhibitors (TKIs), HIF2a inhibitors, anti-angiogenesis agents and immune checkpoint blockade (18, 19). Despite favorable initial responses, most patients relapse and present with drug resistant disease (20, 21). For these patients, there remains an urgent unmet need to develop novel targeted agents.

In this study, we investigated the effects of PRT1419 in preclinical models of PBRM1-mutant and PBRM1-wild-type (PBRM1-WT) ccRCC. We demonstrate that PBRM1 deficiency is associated with increased apoptosis priming and susceptibility to PRT1419. We also show that PRT1419 combines with standard of care (SoC) agents to synergistically inhibit PBRM1-mutant ccRCC growth. We also demonstrate that transient CDK9 inhibition, a clinically validated strategy to inhibit malignant growth by depleting short-lived labile proteins like MCL1 (22, 23), similarly inhibits tumor cell survival and growth in PBRM1-deficient ccRCC but not WT.

## 2 Methods

### 2.1 Cell lines

SNU349 (KCLB 00349) and SNU1272 (KCLB 01272) were purchased from the Korean Cell Line Bank. KMRC1 (JCRB1010) and KMRC-2 (JCRB1011) were purchased from the Japanese Collection of Research Bioresources Cell Bank. A704 (HTB-45), CAKI2 (HTB-47), 769-P (CRL-1933), 786-O (CRL-1932), ACHN (CRL-1611), A498 (HTB-44) and NCI-H1703 (CRL-5889) were purchased from the American Type Cell Collection. OSRC-2 (CSC-C9232W) cells were purchased from Creative Bioarray. Cells were maintained as follows: SNU349, SNU1272, 769-P, 786-O, A498 and NCI-H1703 were cultured in RPMI-1640 (ATCC, 30-2001) supplemented with 10% Fetal Bovine Serum (FBS) (Gibco, 26140-079) and 1X penicillin-streptomycin (Thermofisher, 15140122). KMRC1, KMRC-2 and OSRC-2 were cultured in Dulbecco’s Modified Eagles Medium (DMEM) (Corning, 10-013-CV) supplemented with 10% FBS and 1X penicillin-streptomycin. A704, CAKI2, ACHN and A498 were cultured in Eagle’s Minimum Essential Medium (EMEM) (ATCC, 30-2003) supplemented with 10% FBS and 1X penicillin-streptomycin. All cells were maintained at 37°C in 5% CO_2_ in a humidified incubator.

### 2.2 Cell viability and caspase activation assays

Cells were seeded (2500 cells/well) in 96 well Ultra-Low Adherence (ULA) plates (Corning 7007) and allowed to aggregate into tumor spheroids overnight. Tumor spheroids were treated with varying concentrations of drug using a Tecan D300e Digital Dispenser (Tecan, 30100152). The following commercial compounds were used in this study: Sunitinib (MedChemExpress, HY-10255A, Everolimus (MedChemExpress, HY-10218), Pazopanib (MedChemExpress, HY-10208), Cabozantinib (MedChemExpress, HY-13016) and Belzutifan (MedChemExpress, HY-125840). Following 72h exposure to drug, cell viability was assessed using Promega’s CellTiter-Glo® 3D Cell Viability Assay (Promega, G9681). Cell Viability was calculated as percentage of vehicle treatment. For apoptosis assays, following drug exposure, Caspase 3/7 activity was assessed using Promega’s Caspase-Glo® 3/7 3D Assay system (Promega, G8981). Normalized Caspase 3/7 activity was calculated as a percentage of the difference between a positive control (30 µM PRT1419) and vehicle (DMSO).

### 2.3 RNAi transfection

PBRM1-siRNA (siPBRM1) (Ambion, AM16708) was transfected into 769-P cells via lipofection (Lipofectamine® RNAiMAX, Thermofisher, 13778075) according to manufacturer’s protocol. Briefly, cells were incubated with siPBRM1 and Lipofectamine RNAiMax reagent in antibiotic-free Opti-MEM™ I Reduced Serum Medium (Thermofisher, 31985070) overnight. Transfected cells were then rescued in complete growth media for 1 h prior to being seeded for cell proliferation and protein isolation studies.

### 2.4 PBRM1 knockout cell line generation

A498 cells were electroporated using the NEON transfection system (Invitrogen, MPK10025). Two guideRNA (gRNA) sequences were designed for targeting exon 3 of the PBRM1 gene: 5’- GAAACCACTTCATAATAGTC-3’ and 5’-CAACCAGACTATTATGAAG-3’. Synthetic single gRNAs were ordered from Synthego and recombinant spCas9 protein was purchased from Integrated DNA Technologies. SgRNA and SpCas9 were mixed and allowed to complex at room temperature for approximately 20 minutes before transfection. Cells were seeded two days prior to transfection and allowed to reach approximately 70% confluency. On the day of the transfection, cells were resuspended at a concentration of 5×10^5^ in 100 µL Buffer R from the NEON transfection system and 5 µL of each RNP complex was added. Cells were electroporated using the NEON at 1600 V for three 10 ms pulses and then transferred to a T-25 flask with complete culture media prior to sorting. Transfected cells were sorted 10 cells per well into 96 well plates and transferred to larger plates as they reached confluency. Two PBRM1 KO populations were identified: one with 100% knockout efficiency and one with 55% knockout efficiency.

### 2.5 Protein isolation and immunoblotting

Cell pellets were incubated with RIPA buffer (EMD Millipore, 20-188) supplemented with Pierce protease inhibitor (Thermo Scientific, A32953), on ice for 15 minutes. The resulting cell suspension was centrifuged at 11,000 RPM for 15 minutes and protein supernatant recovered. Protein quantification was performed using Bio-Rad’s Bradford Protein Assay (5000001). Protein lysates were boiled at 95°C for 5 minutes in 1X Laemmli Buffer (1610747) supplemented with 355 mM 2-mercaptoethanol. Denatured proteins were resolved by SDS-PAGE using 4-15% polyacrylamide gels (BioRad, 4561084) and transferred onto 0.2 µm Nitrocellulose (Thermo Scientific, 22860) or 0.45 µm Low Fluorescence PVDF membranes (Bio-Rad, 10026934). Membranes were blocked in 5% non-fat dry milk/TBS-T (BioRad, 1706404 and 97064-338) at room temperature for 1 h. Primary antibody incubations were performed according to supplier recommendations. The following primary antibodies were used in this study: PBRM1 (CST 89123S/E9X2Z), NOXA (CST 147662/D8L7U), GAPDH (CST 97166S/D4C6R), MCL1 (CST 5453S/D35A5) and BCLxL (CST 2764S/54H6). Secondary HRP-linked or Near InfraRed (NIR) antibodies were used to detect protein on the membranes using the LiCOR Odyssey (CLX-2687) or ChemiDoc™ (BioRad, 12003153) imaging systems.

### 2.6 RNA-seq and gene set enrichment analyses

Following siPBRM1 transfection and rescue, 769-P cells were cultured in 3D for 24h. Cells were harvested, centrifuged and the resulting cell pellets stored at -80°C. Frozen cell pellets were transferred to Azenta US, Inc. for RNA isolation, library preparation, indexing, sequencing (Illumina, paired-end 2 x 150 bp) and standard-RNA-seq analysis. Count data was analyzed using DESeq2 and significantly Differentially Expressed Genes (DEGs) (q<0.01) identified. Gene Set Enrichment Analysis (24) was performed on DEGs matching deregulated genes to the MSigDB Hallmark gene set.

### 2.7 Tumor models

Cell line-Derived Xenograft (CDX) studies were performed at Shanghai Medicilon (OSRC-2) and CrownBio (H1703). All studies were performed in accordance with animal research guidelines from the Shanghai Medicilon Inc. Guidelines for Use and Care of Animals Committee or the Institutional Animal Care and Use Committee (IACUC) of CrownBio. For the OSRC-2 model, 1 x 10^6^ OSRC-2 cells were injected into the right flank of 6–9-week-old female BALB/c nude mice (Beijing Vital River Laboratory Animal Technology Co., Ltd.). For the H1703 model, 1 x 10^7^ NCI-H1703 cells were injected into the right front flank region of 6–9-week-old female NOD/SCID mice (GemPharmatech Co., Ltd). When the average tumor size was approximately ~200 mm^3^, mice were randomized. Mice were treated with various agents and tumor size measured using a digital caliper twice a week. Body weight was also assessed biweekly and overall tolerability (body weight loss, lethality, behavioral changes, and clinical signs of adverse treatment-related side effects) monitored daily.

### 2.8 Incucyte tumor growth analyses

KMRC2 cells (PBRM1 mutant, VHL mutant) were seeded in 96w ULA plates (5000 cells/well) and centrifuged briefly (300g, 30 seconds) to induce cell aggregation. The cells were incubated at 37°C overnight to allow complete aggregation into spheroids. Following treatment with PRT1419 and/or Belzutifan, 3D tumor spheroid growth was monitored using Sartorius’ Incucyte® SX1 Live-Cell Analysis System (9600-0031). Apoptosis was assessed via Annexin-V staining (Sartorius, 4641).

## 3 Results

### 3.1 Damaging mutations in PBRM1 are associated with MCL1 dependency and sensitivity to MCL inhibition in ccRCC

**Figure 1:**
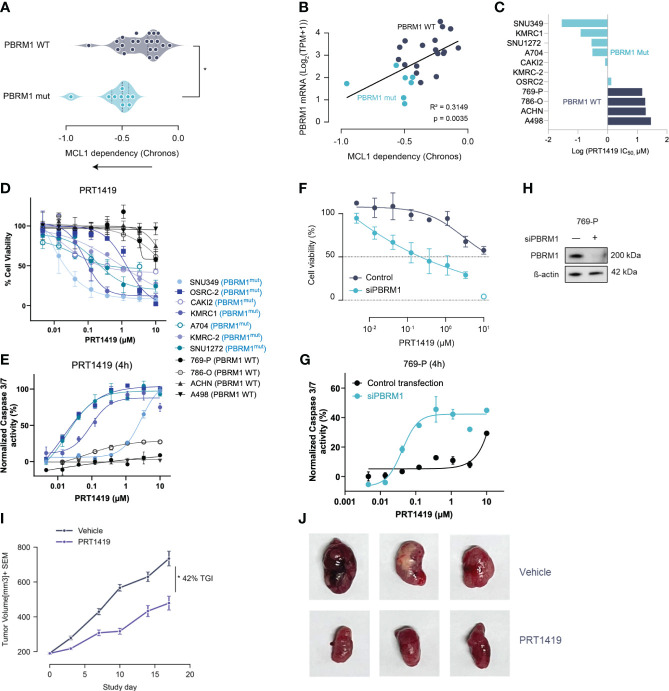
Damaging Mutations in *PBRM1* are associated with MCL1 dependency and sensitivity to MCL inhibition in ccRCC. **(A)** MCL-1 gene dependency analysis in ccRCC cell lines with damaging *PBRM1* mutations compared to WT. Dependency scores (Chronos, 22Q2) were retrieved from DepMap (https://depmap.org) **(B)**. PBRM1 mRNA levels in ccRCC cell lines inversely correlate with MCL1 dependency. *P<0.05, versus WT by t-test. **(C)** Waterfall plot showing half maximal inhibitory concentration (IC50) for PRT1419 in the ccRCC cell line panel. **(D)** Cell Titer-Glo (CTG) assay assessing inhibition of 3D spheroid growth in *PBRM1-*mut and WT ccRCC cell lines following treatment with MCL-1 inhibitor PRT1419 (72h). Values shown are cell viability calculated as percentage of vehicle DMSO control. **(E)** Caspase 3/7 activity assay showing induction of apoptosis following 4h treatment with PRT1419 in *PBRM1*-mut and WT lines. **(F)** CTG assay assessing inhibition of 3D cell growth by PRT1419 in 769-P (*PBRM1* WT) cells following PBRM1 depletion via RNAi. **(G)** Caspase 3/7 activity assays assessing induction of apoptosis by PRT1419 in 769-P (*PBRM1* WT) cells following PBRM1 depletion via RNAi. **(H)** Western blot validating PBRM1 expression following RNAi knockdown in 769-P cells **(I)** OSRC-2 (PBRM1 mutant ccRCC) cell line-derived xenograft (CDX) assessing anti-tumor activity of intravenously administered PRT1419 at 20 mg/kg. Animals were dosed once weekly with PRT1419. Data represented as mean ± SEM. N=8, * P<0.05 by Mann-Whitney U test. **(J)** Representative images of tumors from mice in both vehicle and PRT1419-treated groups at the end of the study.

To identify putative genomic biomarkers of MCL1 sensitivity, we interrogated *MCL1* gene dependency data from genome wide CRISPR/Cas9 cell viability screens using the Broad Institute’s Cancer Dependency Map (DepMap) (10, 11). We focused on identifying driver mutations or alterations that increased dependency on *MCL1*. Our analysis revealed that ccRCC cell lines with inactivating mutations in *PBRM1* had increased dependency on *MCL1* (**Figure 1A**). In the same dataset, low expression of *PBRM1* mRNA also correlated with increased MCL1 dependency (**Figure 1B**). Taken together, these data suggest PBRM1 loss is associated with a functional dependency on MCL1 in ccRCC.

To validate the findings of our genomic screen, we profiled PBRM1 mutant and WT ccRCC cell lines for sensitivity to pharmacological inhibition of MCL1. We had previously described PRT1419, a potent and selective MCL1 inhibitor with robust anti-tumor activity in various preclinical models (25). PRT1419 selectively binds MCL1 with >200 fold selectivity over BCL2 and BCLxL, disrupting MCL1-BIM interactions and inducing caspase-dependent cell death (25). Treatment with PRT1419 for 72 hours potently inhibited 3D cell proliferation and induced apoptotic cell death in a dose-dependent manner in PBRM1-mutant ccRCC cell lines but not WT (**Figures 1C–E**). To validate these findings, we depleted PBRM1 in PBRM1-WT ccRCC cell lines using RNAi and similarly observed increased sensitivity to PRT1419 in a 3D proliferation and apoptosis assay (**Figures 1F–H**). Interestingly, under adherent culture conditions, PRT1419 had no effects on cell growth in both PBRM1 mutant and WT ccRCC, consistent with previous reports evaluating other MCL1 inhibitors (**Supplementary Figure S1A**). RNAi depletion of PBRM1 in adherent cell culture also had no effect on PBRM1 WT ccRCC sensitivity to PRT1419 (**Supplementary Figure S1B**).

To validate the therapeutic feasibility of MCL1 inhibition in ccRCC, we sought to investigate PRT1419 in a cell-line derived xenograft (CDX) model of PBRM1-mutant ccRCC. We have previously shown PRT1419 to be bioavailable when administered orally or intravenously in subcutaneous tumor xenograft models (25, 26). Although most ccRCC cell lines with damaging PBRM1 mutations have poor engraftment rates as subcutaneous tumors in nude mice (27), we identified one cell line, OS-RC-2, which harbors a missense mutation in the *PBRM1* bromodomain as a potential system to model PBRM1 loss *in vivo* (27). Importantly, OS-RC-2 lacks PBRM1 protein expression and can be transplanted into nude mice, generating tumors with histopathological features which closely resemble clinical ccRCC (28). OS-RC-2 cells were inoculated subcutaneously into 6–9-week-old female BALB/c nude mice. When the tumors were ~200 mm^3^, mice were randomized into two groups. Animals were either treated with vehicle or 20 mg/kg of PRT1419 administered intravenously, once weekly for three weeks. We observed 42% Tumor Growth Inhibition (TGI) in response to PRT1419 treatment (**Figures 1I, J**). PRT1419 dosing was well tolerated with no notable body weight loss or behavioral changes. Altogether these findings suggest PBRM1 loss in ccRCC is associated with MCL1 dependency and sensitivity to MCL1 inhibition.

### 3.2 PBRM1 loss in ccRCC is associated with pro-apoptotic signaling, priming cells for cell death following MCL1 inhibition

**Figure 2:**
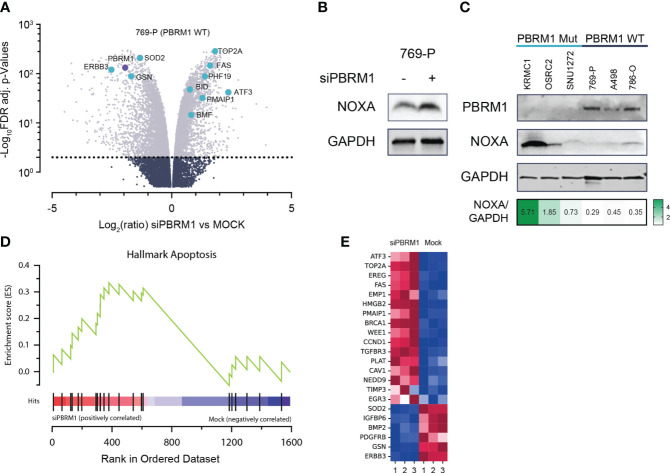
PBRM1 loss in ccRCC is associated with pro-apoptotic signaling, thereby priming cells for cell death following MCL1 inhibition. **(A)** Volcano plots of global RNA expression data in 769-P (PBRM1 WT ccRCC) cells following RNAi-mediated PBRM1 depletion and 24h spheroid culture; genes mediating apoptosis are highlighted in blue. **(B)** Western blot showing increased expression of pro-apoptotic MCL1 inhibitor, NOXA (*PMAIP1*), following RNAi-mediated depletion of PBRM1 and 24h 3-D culture. **(C)** Western blot showing increased endogenous expression of NOXA in *PBRM1-*mut ccRCC cell lines cultured as spheroids. **(D, E)** Unbiased Gene Set Enrichment Analysis (GSEA) analysis (MSigDB Hallmark geneset) showing increased expression of a pro-apoptosis gene signature in ccRCC following RNAi-mediated depletion of PBRM1.

To elucidate the mechanistic link between PBRM1 loss and increased MCL1 dependency, we performed whole transcriptome analyses, assessing gene expression changes in PBRM1 WT ccRCC cells following RNAi depletion of PBRM1. Using RNA-seq in 769-P cells, we observed significant deregulation of gene expression following PBRM1 siRNA knockdown and spheroid culture (**Figure 2A**). Several pro-apoptosis factors were upregulated following PBRM1 depletion including *BMF*, *BID*, *PMAIP1* (NOXA) and *FAS*. We were particularly interested in the observed upregulation of NOXA, a pro-apoptotic BH3-only protein that selectively binds MCL1 over other Bcl-2 family proteins, targeting it for degradation (29, 30). Increased expression of NOXA has previously been described as a biomarker for sensitivity to MCL1 inhibition (31, 32). Using immunoblotting, we confirmed increased NOXA protein expression following PBRM1 siRNA depletion in 769-P cells, corroborating the findings of the transcriptome analysis (**Figure 2B**). In an extended panel of ccRCC cell lines, we similarly observed increased endogenous NOXA expression in PBRM1 mutant ccRCC cells compared to WT (**Figure 2C**). Strikingly, despite increased sensitivity to MCL1 inhibition, PBRM1 mutant ccRCC cell lines had lower endogenous levels of MCL1 (**Supplementary Figure S2A**). MCL1/BCLxL ratios, which have previously been described to predict sensitivity to MCL1 inhibitors (33) were also paradoxically lower in PBRM1 mutant ccRCC cells compared to WT (**Supplementary Figure S2B**).

To further characterize the link between PBRM1-loss-associated gene deregulation and increased MCL1i sensitivity, we performed unbiased gene set enrichment analysis (GSEA) on significantly differentially expressed genes (DEGs) from the RNA-seq analysis. Matching the DEGs to the MSigDB Hallmark geneset in GSEA, we identified several key oncogenic pathways deregulated following PBRM1 depletion in ccRCC. Notably, the HALLMARK_APOPTOSIS pathway, a gene signature associated with increased propensity for apoptosis, was found to be enriched in PBRM1 depleted cells (**Figures 2D, E**). Taken together, these results suggest PBRM1 depleted ccRCC cells exist in a pro-apoptotic, “primed” state, increasing their susceptibility to cell-death-targeted agents like MCL1 inhibitors.

### 3.3 MCL1 inhibition synergizes with standard of care agents in PBRM1-mutant ccRCC

**Figure 3:**
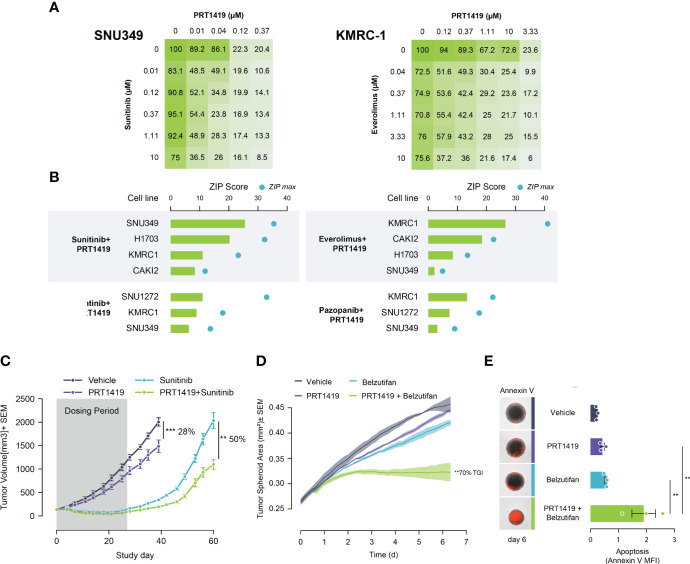
PRT1419 synergizes with Standard of Care (SoC) agents in PBRM1-mutant ccRCC. **(A)** CellTiter-Glo assay demonstrating synergistic inhibition of spheroid growth in *PBRM1*-mutant cell lines following treatment with PRT1419 and SoC agents: Sunitinib and Everolimus. Values shown are cell viability calculated as percentage of DMSO control. **(B)** ZIP synergy scores from synergy proliferation assays were computed using SynergyFinder 2.0 (https://synergyfinder.fimm.fi/) Cells were co-treated with PRT1419 and Everolimus/Sunitinib/Cabozantinib/Pazopanib for 72h. **(C)** H1703 CDX (PBRM1 protein loss NSCLC) model assessing anti-tumor activity of intravenously administered PRT1419 (20 mg/kg) in monotherapy and in combination with Sunitinib (administered orally). Animals were dosed with PRT1419 once a week and/or Sunitinib (10 mg/kg) daily. Data represented as mean ± SEM. N=8, **P<0.001, ***P<0.001 by unpaired t test **(D, E)** Incucyte-based measurement of spheroid growth and apoptosis (Annexin V) in KMRC2 (VHL protein loss, PBRM1 mutant) following treatment with PRT1419 (0.5 µM) and/or Belzutifan (HIF2ɑ inhibitor, 1 µM) for 6 days. Data represented as mean ± SEM. N=3, ** P<0.01 by **(A)** one-way ANOVA.

Despite good initial responses, most patients with metastatic ccRCC develop resistance to SoC agents (20, 21). Increased expression of Bcl-2 pro-survival factors like MCL1 and BCLxL have previously been described as resistance mechanisms to ccRCC SoC agents like TKIs, and mTORCi (34, 35). We hypothesized that inhibiting MCL1 would potentiate the anti-tumor effect of these agents, creating a unique clinical opportunity to combine both agents for improved responses. To test this, we co-treated PBRM1 mutant ccRCC cells with varying doses of PRT1419 and Everolimus (mTORCi) or Sunitinib (TKI) for 72h and assessed their combined effect on 3D cell proliferation. The combined effect on cell viability was interrogated for synergism using the Zero Interaction Potency (ZIP) model. We observed synergistic inhibition of spheroid growth in PBRM1-mutant ccRCC cell lines following co-treatment with PRT1419 and Sunitinib or Everolimus (**Figures 3A, B**). Expanding our analysis to other TKIs including Cabozantinib and Pazopanib, we similarly observed synergistic anti-proliferation activity (**Figure 3B**). No synergy was observed between PRT1419 and both Everolimus and Sunitinib in 769-P (PBRM1 WT) (**Supplementary Figure S3**). To validate these results *in vivo*, we assessed the effect of co-administration of PRT1419 and Sunitinib on H1703 xenograft growth. H1703 is a lung cancer cell line with PBRM1 protein loss (11) that has been extensively used to characterize responses to Sunitinib in monotherapy and in combination with various agents (36–39). Female NOD/SCID mice were injected subcutaneously with H1703 cells. When the tumors were ~200 mm^3^, animals were dosed with 20 mg/kg of PRT1419 intravenously, once a week and/or 10 mg/kg of Sunitinib, orally, daily. Following 3 weeks of dosing, we observed inhibition of tumor growth by both PRT1419 and Sunitinib (28% and 100% respectively) (**Figure 3C**). Following the conclusion of dosing, rapid tumor re-growth was observed in animals treated with Sunitinib alone. Co-administration of PRT1419 potently repressed tumor re-growth in the combination groups (50% tumor re-growth inhibition) suggesting a more durable therapeutic response.

We next sought to characterize the potential combined anti-tumor effect of PRT1419 and HIF2α inhibitor Belzutifan in PBRM1 mutant ccRCC. Belzutifan was recently approved for the treatment of von Hippel–Lindau (VHL) loss renal cell carcinoma (18). To model the anti-tumor effects of HIF2α inhibitors *in vitro*, we developed a hypoxia-simulating spheroid growth assay. When grown as compact spheroids, tumor cells develop a hypoxic core which recapitulates the hypoxic intratumoral environment of ccRCC, inducing expression of hypoxia inducible factors like HIF2α (40, 41). We observed potent, combinatorial inhibition of spheroid growth by PRT1419 and Belzutifan in PBRM1 mutant, VHL-null ccRCC cell line KRMC-2 (**Figure 3D**). The combined effect on spheroid growth also coincided with increased apoptotic cell death as assessed by Annexin V staining (**Figure 3E**). Taken together, these results suggest PRT1419 potentiates the anti-tumor effects of multiple classes of ccRCC SoC agents *in vitro* and *in vivo*.

### 3.4 Transient CDK9 inhibition similarly demonstrates antitumor activity in PBRM1-loss ccRCC and synergizes with SoC agents

**Figure 4:**
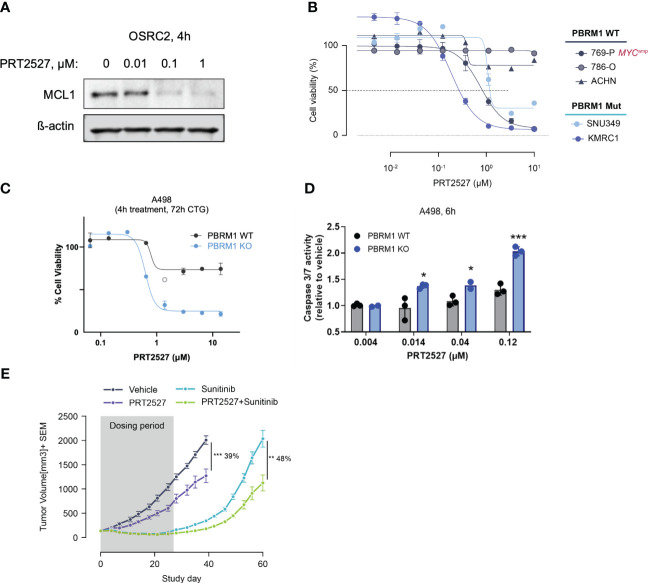
Transient CDK9 inhibition demonstrates antitumor activity in PBRM1-loss ccRCC and synergizes with SoC agents. **(A)** Western blot assessing MCL1 expression following 4h treatment with varying doses of PRT2527. **(B)** CellTiter-Glo assay assessing inhibition of spheroid growth in *PBRM1-*mutant and WT ccRCC cell lines following brief treatment with CDK9 inhibitor PRT2527. Cells grown as spheroids were treated for 4h, then cultured in drug-free media for 48h **(C, D)** CTG and caspase 3/7 activity assay assessing inhibition of 3D cell growth and induction of apoptosis in A498 PBRM1 WT and A498 PBRM1 KO cells following treatment with PRT2527. **(E)** Intravenously administered PRT2527 combines with Sunitinib (dosed orally) to repress tumor growth in a PBRM1-protein loss NSCLC (NCI-H1703) cell line-derived xenograft model. Animals were dosed with PRT2527 (30 mg/kg) once a week (twice in one day) and/or Sunitinib (10 mg/kg) daily. Data represented as mean ± SEM. N=8, *P <0.05, **P<0.001, ***P<0.001 by unpaired t test.

CDK9 is a master regulator of transcription that modulates transcription elongation via phosphorylation of RNA polymerase II (22, 23) and CDK9 inhibition has been extensively evaluated in various malignancies (42). We and several others have recently shown that short-term inhibition of CDK9 depletes short-lived transcripts and labile proteins such as MCL1 and MYC to promote cancer cell death (43–45). We reasoned that by depleting MCL1, transient CDK9 inhibition would similarly be efficacious in PBRM1-mutant ccRCC. To test this, we treated PBRM1 mutant and WT ccRCC cells with PRT2527, a selective CDK9 inhibitor. Short-term (4h) treatment with PRT2527 potently depleted MCL1 protein in OSRC-2 cells (**Figure 4A**). We then assessed the effect of PRT2527-mediated MCL1 depletion on ccRCC spheroid growth. Short-term treatment (4h) with PRT2527 potently inhibited spheroid growth in PBRM1 mutant ccRCC cell lines but not WT (**Figure 4B**). Interestingly, 769-P, a PBRM1-WT cell line, displayed pronounced sensitivity to CDK9 inhibition. 769-P is a MYC-amplified cell line, a phenotype that generally independently confers sensitivity to transient CDK9 inhibition (46). To validate the link between PBRM1 loss and sensitivity to CDK9 inhibition, we developed a *PBRM1* knock-out (KO) cell line using A498, a PBRM1 WT ccRCC cell line as background. Short-term (4h) treatment with PRT2527 potently inhibited spheroid cell growth and induced apoptosis in *PBRM1 KO* A498 cells but not WT (**Figures 4C, D**). *In vivo*, once weekly administration of PRT2527 (30 mg/kg) potently inhibited tumor growth in a H1703 (PBRM1-protein-deficient lung cancer) CDX model (39% TGI) (**Figure 4E**). In the same manner as PRT1419, co-treatment with Sunitinib inhibited tumor re-growth (48% tumor re-growth inhibition) suggesting a more sustained therapeutic effect (**Figure 4E**). Altogether, these results suggest MCL1 inhibition, either directly or indirectly through CDK9 inhibition, has potent anti-tumor activity in PBRM1-deficient ccRCC.

## 4 Discussion

Anti-apoptosis factors including MCL1, BCL2, BCLxL and BFL1 are crucial mediators of tumorigenesis and resistance to therapy in various cancers (1, 2, 47). Pharmacological inhibition of these proteins, notably BCL2, has shown remarkable clinical efficacy and improved patient outcomes in several hematologic cancers (48–50). In solid cancers, however, clinical responses to BCL2 family-targeted agents have been mediocre (51). To improve patient outcomes and increase therapeutic indices, biomarker-driven patient stratification strategies are paramount. Here we demonstrate that PBRM1 loss in ccRCC is associated with increased sensitivity to direct and indirect MCL1 inhibition, highlighting MCL1 and CDK9 inhibitors like PRT1419 and PRT2527 as potential therapeutic options for this genetically defined patient subset with unmet need.

PBRM1 is a crucial chromatin-targeting subunit of mammalian pBAF (SWI/SNF-B) complexes (12, 13). In several cancers, notably ccRCC, PBRM1 functions as a tumor suppressor, inhibiting malignant tumor transformation and growth (12, 52). Deleterious *PBRM1* alterations in ccRCC are associated with increased cell proliferation, motility, stemness and resistance to apoptosis (12, 52–54). Despite the strong correlation between PBRM1 loss and increased tumorigenicity, the predictive value of PBRM1 loss on therapeutic outcomes in ccRCC patients remains unclear. PBRM1 loss is associated with increased relapse and recurrence in ccRCC (55, 56). Paradoxically, however, in relapsed disease, PBRM1 loss has been associated with both improved and dampened responses to anti-angiogenesis agents, mTOR inhibitors and immune checkpoint blockade (56–59). Further retrospective and prospective analyses of clinical trial data characterizing the effects of deleterious *PBRM1* alterations on depth and duration of response to these agents is necessary.

The tumorigenic effects of PBRM1 loss in ccRCC cells are more pronounced in spheroid or anchorage-independent growth conditions (54, 60) which more accurately recapitulate the morphology and clonal heterogeneity of clinical ccRCC (61, 62). Corroborating this, we found that PBRM1 loss conferred increased sensitivity to MCL1 inhibition in 3D culture but not in 2D. We demonstrated that PBRM1 depletion in ccRCC cells cultured in 3D led to increased expression of a pro-apoptotic gene signature, suggesting increased apoptosis “priming” and susceptibility to apoptosis-inducing agents like MCL1 inhibitors. Previous studies have similarly shown that upregulation of pro-apoptotic gene signatures primes cancer cells for apoptosis and could serve as a biomarker for increased sensitivity to inhibition of Bcl-2 family members (63–66). A recent report further demonstrated that a subset of ccRCC characterized by a mesenchymal transcriptional profile is highly dependent on BCLxL, another Bcl-2 family member (67). Interestingly, MCL1 expression was lower in PBRM1 loss ccRCC compared to WT. Lower expression of MCL1 has previously been associated with increased sensitivity to clinical MCL1 inhibitors (68). In a PBRM1-loss context, it is plausible that reduced MCL1 levels might similarly be associated with increased sensitivity to PRT1419.

Clinically, MCL1 inhibitors are being explored in combination with various targeted agents including TKIs, mitotic inhibitors, autophagy inhibitors and anti-angiogenesis agents (69, 70). Because increased MCL1 expression is frequently associated with resistance to multiple classes of therapeutic agents, MCL1 inhibitors make for ideal combination partners to improve and prolong therapeutic responses. Along these lines, we showed that PRT1419 and PRT2527 combine with and enhance the anti-tumor effects of TKIs (Sunitinib, Pazopanib, Cabozantinib), mTOR inhibitors (Everolimus) and HIF2a inhibitors (Belzutifan). Clinical exploration of these combinations could yield improved outcomes for ccRCC patients with therapy resistant disease.

The clinical exploration of MCL1 inhibitors has been impeded by dose-limiting adverse events, including potential cardiac toxicity (69–72). However, biomarker-driven patient selection strategies to improve therapeutic indices at lower dose levels are yet to be explored. Our work highlights one such potential strategy in a genetically defined patient subset with unmet need. Combined with emerging next-generation cardiac-sparing MCL1 inhibitors (73, 74), such strategies could enable effective and safe use of MCL1 inhibitors in the clinic.

## Data availability statement

Datasets are available on request: The raw data supporting the conclusions of this article will be made available by the authors, without undue reservation.

## Ethics statement

Ethical approval was not required for the studies on humans in accordance with the local legislation and institutional requirements because only commercially available established cell lines were used. The animal study was approved by Shanghai Medicilon Inc. Guidelines for Use and Care of Animals Committee; Institutional Animal Care and Use Committee (IACUC) of CrownBio. The study was conducted in accordance with the local legislation and institutional requirements.

## Author contributions

NF: Conceptualization, Data curation, Formal analysis, Investigation, Methodology, Project administration, Supervision, Validation, Writing – original draft, Writing – review & editing. AS: Conceptualization, Investigation, Methodology, Writing – review & editing. SM: Investigation, Writing – review & editing. AG: Investigation, Writing – review & editing. SR: Investigation, Writing – original draft. BT: Investigation, Writing – review & editing. DH: Writing – review & editing. PS: Project administration, Supervision, Writing – review & editing. NB: Conceptualization, Methodology, Project administration, Supervision, Writing – review & editing.

## Supplementary material

Supplementary Figure 1 PBRM1 loss predicts sensitivity to MCL1 inhibition in spheroid culture but not adherent culture. **(A)** CTG assay assessing PRT1419 inhibition of growth in PBRM1-mutant ccRCC cell lines cultured as 3-D spheroids or 2-D monolayers. Values shown are cell viability calculated as percentage of DMSO control. **(B)** CTG assay assessing PRT1419 inhibition of growth following RNAi-mediated depletion of PBRM1 and 2D culture in 769-P cells (PBRM1 WT).

Supplementary Figure 2 MCL1/BCLxL ratio does not predict sensitivity to MCL1 inhibition in ccRCC. **(A)** Western blot showing endogenous expression of MCL1 and BCLxL in ccRCC cell lines cultured in 3D or 2D. **(B)** qPCR showing *MCL1/BCLxL* ratio, computed as ratio of delta Ct values for both genes, in ccRCC cell lines cultured in 3D.

Supplementary Figure 3 No synergy observed between PRT1419 and Everolimus/Sunitinib in PBRM1 WT cell line 769-P. Cell Titer-Glo assay measuring cell viability in 769-P cells treated with PRT1419 and Everolimus/Sunitinib for 72h. Values shown are cell viability calculated as percentage of vehicle (DMSO) control.
