# Supplementary figures and images for: PBRM1 loss is associated with increased sensitivity to MCL1 and CDK9 inhibition in clear cell renal cancer
Source: Front Oncol. 2024 Feb 2;14:1343004. doi: 10.3389/fonc.2024.1343004 (PMC10869502; doi:10.3389/fonc.2024.1343004)

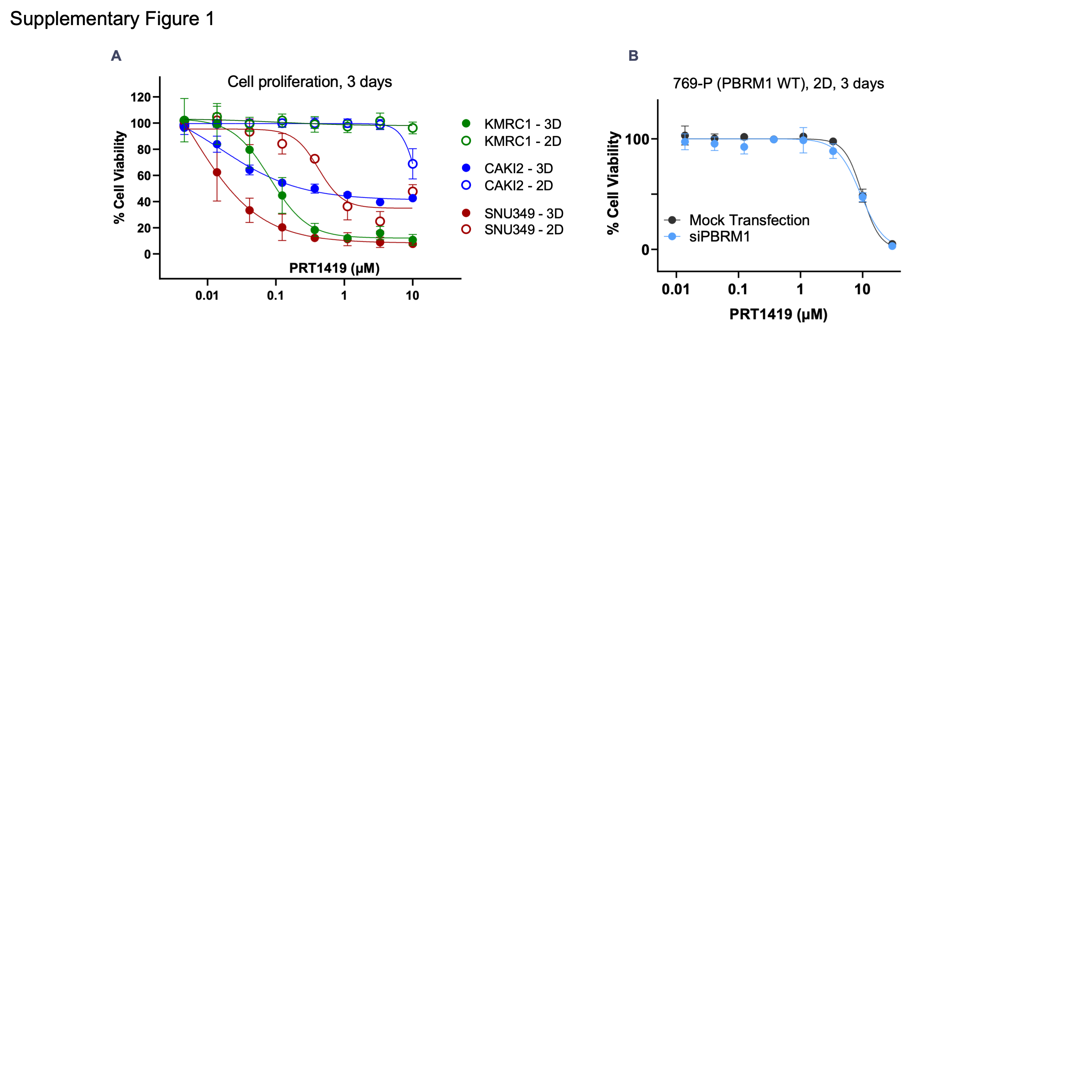

Supplement: Supplementary Figure 1 — PBRM1 loss predicts sensitivity to MCL1 inhibition in spheroid culture but not adherent culture. (A) CTG assay assessing PRT1419 inhibition of growth in PBRM1-mutant ccRCC cell lines cultured as 3-D spheroids or 2-D monolayers. Values shown are cell viability calculated as percentage of DMSO control. (B) CTG assay assessing PRT1419 inhibition of growth following RNAi-mediated depletion of PBRM1 and 2D culture in 769-P cells (PBRM1 WT). [file Image_1.tif]

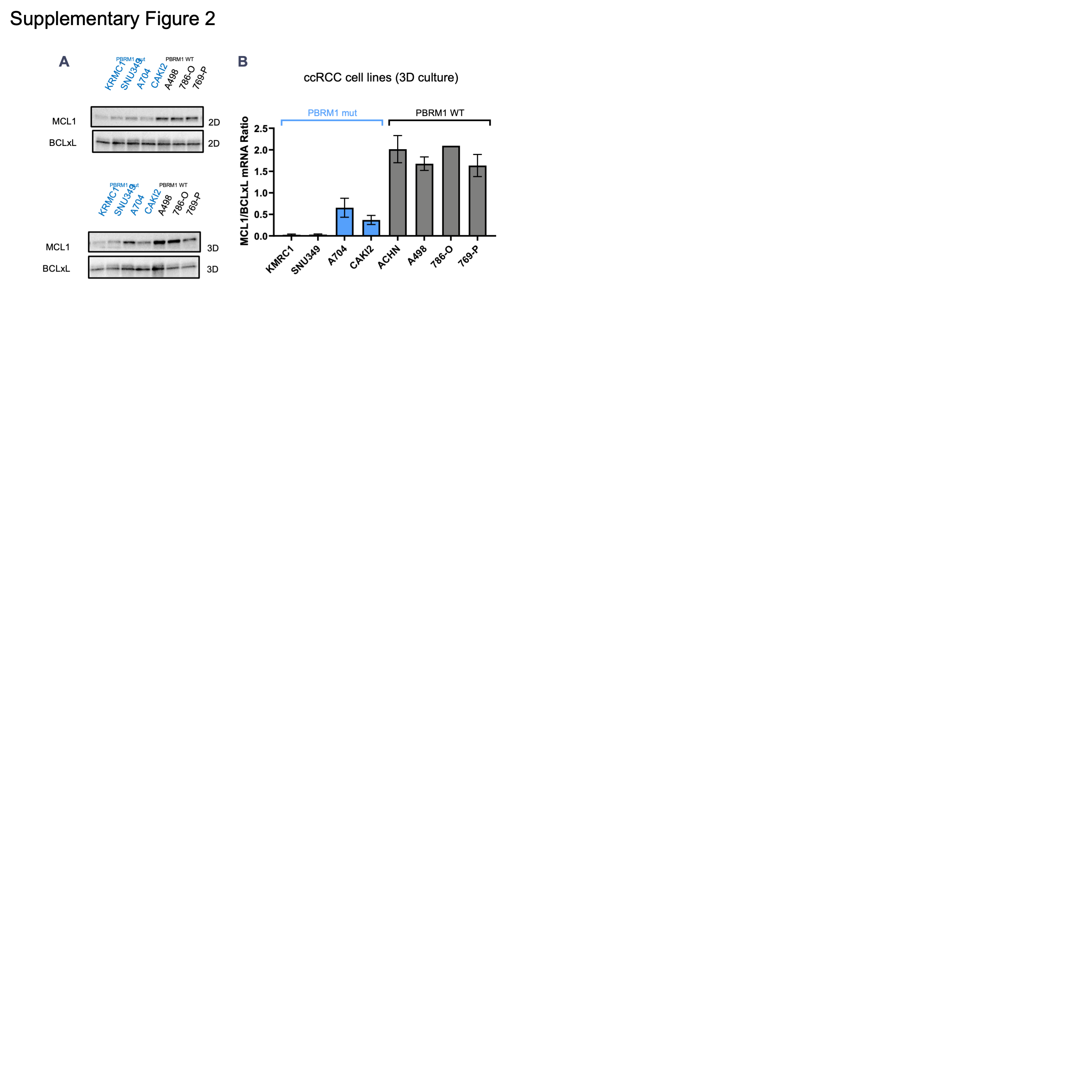

Supplement: Supplementary Figure 2 — MCL1/BCLxL ratio does not predict sensitivity to MCL1 inhibition in ccRCC. (A) Western blot showing endogenous expression of MCL1 and BCLxL in ccRCC cell lines cultured in 3D or 2D. (B) qPCR showing MCL1/BCLxL ratio, computed as ratio of delta Ct values for both genes, in ccRCC cell lines cultured in 3D. [file Image_2.tif]

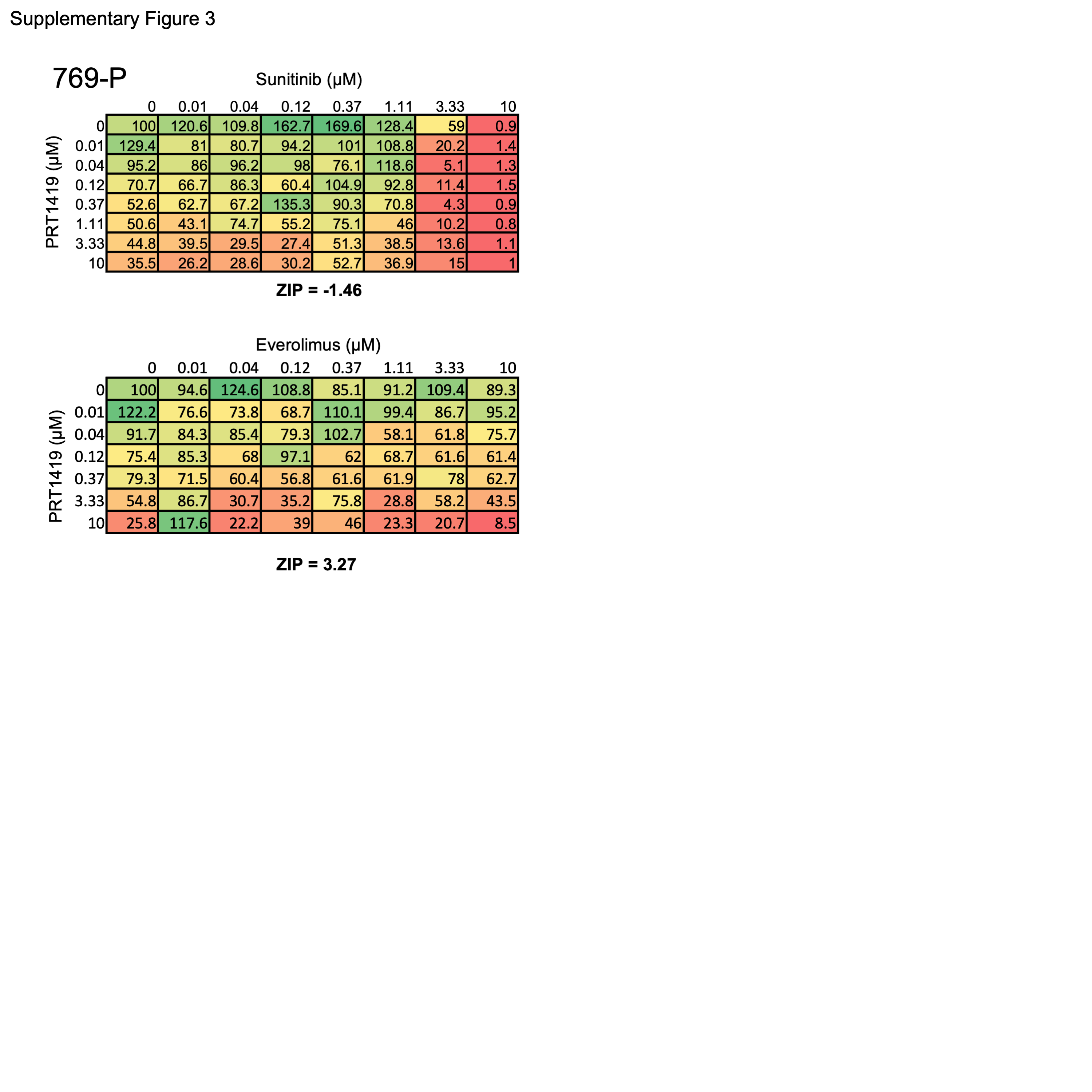

Supplement: Supplementary Figure 3 — No synergy observed between PRT1419 and Everolimus/Sunitinib in PBRM1 WT cell line 769-P. Cell Titer-Glo assay measuring cell viability in 769-P cells treated with PRT1419 and Everolimus/Sunitinib for 72h. Values shown are cell viability calculated as percentage of vehicle (DMSO) control. [file Image_3.tif]
